# Biomechanical Evaluation and the Assisted 3D Printed Model in the Patient-Specific Preoperative Planning for Thoracic Spinal Tuberculosis: A Finite Element Analysis

**DOI:** 10.3389/fbioe.2020.00807

**Published:** 2020-07-17

**Authors:** Bingjin Wang, Wencan Ke, Wenbin Hua, Xianlin Zeng, Cao Yang

**Affiliations:** Department of Orthopaedics, Union Hospital, Tongji Medical College, Huazhong University of Science and Technology, Wuhan, China

**Keywords:** finite element analysis, 3D printed model, thoracic spinal tuberculosis, preoperative planning, spinal fixation, biomechanics

## Abstract

Posterior fixation is superior to anterior fixation in the correction of kyphosis and maintenance of spinal stability for the treatment of thoracic spinal tuberculosis. However, the process of selecting the appropriate spinal fixation method remains controversial, and preoperative biomechanical evaluation has not yet been investigated. In this study, we aimed to analyze the application of the assisted finite element analysis (FEA) and the three-dimensional (3D) printed model for the patient-specific preoperative planning of thoracic spinal tuberculosis. An adult patient with thoracic spinal tuberculosis was included. A finite element model of the T7−T11 thoracic spine segments was reconstructed to analyze the biomechanical effect of four different operative constructs. The von Mises stress values of the implants in the vertical axial load and flexion and extension conditions under a 400-N vertical axial pre-load and a 10-N⋅m moment were calculated and compared. A 3D printed model was used to describe and elucidate the patient’s condition and simulate the optimal surgical design. According to the biomechanical evaluation, the patient-specific preoperative surgical design was prepared for implementation. The anterior column, which was reconstructed with titanium alloy mesh and a bone graft with posterior fixation using seven pedicle screws (M+P) and performed at the T7–T11 level, decreased the von Mises stress placed on the right rod, T7 pedicle screw, and T11 pedicle. Moreover, the M+P evaded the left T9 screw without load bearing. The 3D printed model and preoperative surgical simulation enhanced the understanding of the patient’s condition and facilitated patient-specific preoperative planning. Good clinical results, including no complication of implants, negligible loss of the Cobb angle, and good bone fusion, were achieved using the M+P surgical design. In conclusion, M+P was recommended as the optimal method for preoperative planning since it enabled the preservation of the normal vertebra and prevented unnecessary internal fixation. Our study indicated that FEA and the assisted 3D printed model are tools that could be extremely useful and effective in the patient-specific preoperative planning for thoracic spinal tuberculosis, which can facilitate preoperative surgical simulation and biomechanical evaluation, as well as improve the understanding of the patient’s condition.

## Introduction

Spinal tuberculosis has a severe impact on health worldwide, particularly in developing countries. In southwest China, thoracic spinal tuberculosis was found to occur in approximately 43.6% of spinal sites (Yao et al., 2017). Surgical treatment is necessary to clear the lesion, relieve spinal cord and nerve compression, and reconstruct spinal stability. Surgical treatment consists of the anterior approach, the posterior approach, and the combined anterior and posterior surgery. However, the optimal surgical approaches are still controversial. Previous scholars contend that posterior fixation is superior to anterior fixation in the correction of kyphosis and maintenance of spinal stability (Hassan and Elmorshidy, 2016; Wang et al., 2017; Wu et al., 2018). Furthermore, posterior fixation involves the unaffected vertebrae two levels above and below the affected site (D’souza et al., 2017). It had been recommended that surgical indications for the posterior-only approach include the lesion being confined to less than three adjacent segments, only one or two vertebrae requiring surgery, and complete debridement being achieved through the posterior-only approach (Wu et al., 2018). However, the damaged posterior column has the potential risk of developing spinal instability (Zhang et al., 2013). Even though the screw could be placed in the undamaged part of the diseased vertebrae, the biomechanical stability may be uncertain (Zhang et al., 2013; Liang et al., 2019). Therefore, based on preoperative biomechanical evaluation, selecting the optimal surgical approach can guarantee the best long-term curative effect. However, due to the lack of clinical biomechanical evidence on the posterior approach for thoracic spinal tuberculosis, the process of selecting the appropriate spinal fixation remains controversial. Moreover, preoperative biomechanical evaluation is important for selecting effective surgical approach and ensuring postoperative spinal stability.

Finite element analysis (FEA) has been widely used to evaluate the biomechanical effect of surgeries (Ottardi et al., 2016; Wang B. et al., 2019; Wong et al., 2020). FEA can also facilitate the biomechanical assessment of spinal disease, such as degeneration, compression fracture, and scoliosis (Natarajan and Andersson, 2017; Dupuis et al., 2018; Nakashima et al., 2018). In addition, the range of motion (ROM), intradiscal pressure (IDP), facet joint stress, and internal fixation device stress were calculated and analyzed to evaluate the biomechanical effect of different surgical approaches. Several previous studies indicated that FEA enabled the evaluation of the ROM and IDP of the adjacent segments to predict the risk factors of adjacent segment degeneration after cervical or lumbar surgeries (Ke et al., 2019; Wang B. et al., 2019; Hua et al., 2020; Wong et al., 2020). However, the studies only focused on the postoperative effect of internal fixation to evaluate the biomechanical advantages and disadvantages of surgical treatment. Preoperative planning for spinal diseases such as thoracic spinal tuberculosis has not yet been evaluated. It had been reported that the maximal stress of internal implant was related to the risk of postoperative complications, such as screw breakage and mesh or cage subsidence (Zhang M. et al., 2016; Fan et al., 2019). In the present study, a finite element model of the thoracic vertebrae was designed to evaluate the biomechanical effects of the different methods of anterior column reconstruction and posterior fixations and to determine which type of posterior fixation provides effective spinal stability and less stress on the internal fixation devices. Because of the individual variations in spinal damage, it is plausible that it may be possible to establish patient-specific models for preoperative planning.

In addition, the three-dimensional (3D) printed models have been applied in the preoperative planning of pedicle screw placement for middle-upper thoracic trauma (Xu et al., 2017). This technique may also aid in the performance of complex spinal operations, such as resection of primary cervical spinal tumors and thoracic tumors (Xiao et al., 2016; Choy et al., 2017). Additionally, the patient-specific navigational templates have been applied to pedicle screw placements (Sugawara et al., 2013; Kaneyama et al., 2015; Chen et al., 2016; Takemoto et al., 2016). The 3D printed models could help surgeons plan operations and enhance the understanding of patients’ conditions (Liu et al., 2020). In present study, a 3D printed thoracic vertebrae model was applied to simulate debridement and anterior column reestablishment and to elucidate the patient’s condition and surgical approach.

In the current study, a preoperative finite element model of the thoracic vertebrae was established, four surgical constructs were simulated and the internal fixation device stress under flexion and extension conditions was analyzed and compared in purpose of determining effective surgical construct. In addition, an assisted 3D printed model was reconstructed to describe the patient’s condition and simulate optimum surgical construct, in order to enhance the understanding of disease and surgical approach preoperatively. The aim of this study was to evaluate the biomechanical effects of different preoperative surgical constructs and assess whether FEA and an assisted 3D printed model was useful in patient-specific preoperative planning for thoracic spinal tuberculosis.

## Materials and Methods

### Reconstruction of the Thoracic Vertebrae Finite Element and 3D Printed Models

**FIGURE 1:**
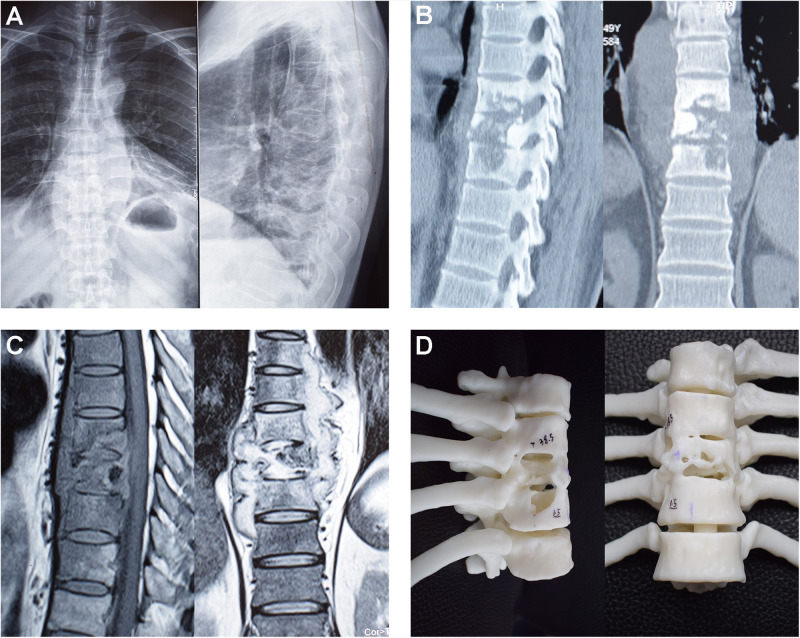
Preoperative imaging results of the patient with thoracic spinal tuberculosis. **(A)** preoperative anteroposterior and lateral radiography; **(B)** preoperative CT images; **(C)** preoperative MRI; **(D)** preoperative 3D printed model.

A 48-year-old man with severe back pain for 1 month was included in this study. He was diagnosed as thoracic spinal tuberculosis and was admitted to the hospital for surgery. The diagnosis of tuberculosis was based on the clinical symptoms, laboratory examination, and imaging results including anteroposterior and lateral radiography, computed tomography (CT), and magnetic resonance imaging (Figures 1A–C). A finite element model of the T7–T11 thoracic spine segments was developed from CT images with a slice thickness of 0.625 mm, obtained from the patient. CT images of the patient were stored in DICOM format. The geometric model of the thoracic spine was constructed using Mimics 20.0 (Materialize, Leuven, Belgium), and the mesh structures of the model were prepared using HyperMesh (Altair Technologies Inc., Fremont, CA, United States). The thoracic spinal models were imported into ANSYS (ANSYS, Ltd., Canonsburg, PA, United States) to perform the biomechanical analysis.

**FIGURE 2:**
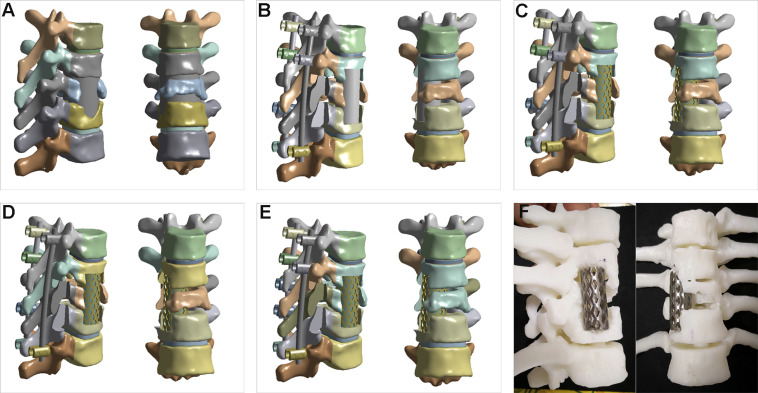
Thoracic vertebrae finite element models and surgery simulation. **(A)** preoperative finite element model of the T7−T11 thoracic spine segments; **(B)** anterior column reconstructed with bone graft and posterior fixation with initial seven pedicle screws (B+P); **(C)** anterior column reconstructed with mesh and posterior fixation with initial seven pedicle screws (M+P); **(D)** right T8 pedicle screw removed from M+P construct (M++P-−T8); **(E)** left T9 pedicle screw added into M+P construct (M+P+T9); **(F)** surgical simulation in 3D printed model.

This finite element model included the cortical bone (thickness, 1 mm), cancellous bone, posterior elements, and intervertebral discs. A closed surface of cortical bones assigned to 8-node solid hexahedral elements. Cancellous bone and posterior elements were assigned to 4-node solid tetrahedral elements. The intervertebral discs were separated into annulus fibrosus, nucleus pulposus, and superior and inferior endplates (thickness, 0.5 mm). The intervertebral discs were composed of 56% annulus fibrosus surrounding 44% incompressible nucleus pulposus. The fluid-like behavior of the annulus ground substance and nucleus pulposus was simulated using a hyperelastic Mooney-Rivlin formulation according to previous study (Dreischarf et al., 2014; Ambati et al., 2015). Fibers of annulus fibrosus were embedded on the annulus ground substance, with these fibers acting at 30° and 150° from the horizontal in eight layers (Ambati et al., 2015). Young modulus of these fibers was assigned to decrease from the outermost layer (550 MPa) to the innermost layer (358 MPa). The endplates were connected to the vertebrae by sharing common nodes on the interfaces. The facet joints were composed of the superior and inferior articular processes and were simulated with surface-to surface contact with friction. A finite sliding interaction was defined and friction coefficient of 0.1 was assigned. Bonded contact was assigned between other components. Seven ligaments, including the anterior longitudinal ligament, posterior longitudinal ligament, ligamentum flavum, capsular ligament, interspinous ligament, intertransverse ligament, and supraspinal ligament, were modeled as hypoelastic, tension-only, truss elements. The material properties for bone, intervertebral discs, and ligaments were derived from the ligament stiffness data of previous studies and are shown in Table 1 (Fagan et al., 2002; Jones and Wilcox, 2008; Wang B. et al., 2019; Hua et al., 2020). The finite element model of the T7−T11 thoracic spine segments is shown in Figure 2A. The reconstructed 3D model of the T7−T11 thoracic spine segments according to preoperative CT images was imported into a 3D printer, and a 1:1 resin model was printed out (Figure 1D).

**TABLE 1:** Material properties of the finite element models.

Component/materials	Element type	Young’s modulus E (MPa)	Poisson’s ratio	Cross section area (mm^2^)	Element number
Cortical bone	C3D8	12000	0.3	−	35682
Cancellous bone	C3D4	100	0.2	−	102294
Posterior elements	C3D4	3500	0.25	−	82290
Endplate	C3D8	1000	0.3	−	21451
Nucleus pulposus	C3D8H	Hyperplastic (Mooney-Rivlin) 1 (C_1_ = 0.12, C_2_ = 0.03)	0.499	−	16896
Annulus ground substance	C3D8	Hyperplastic (Mooney-Rivlin) 1.75 (C_1_ = 0.2333, C_2_ = 0.0583)	0.45	−	21434
Annulus fibrosus	T3D2	358–550	0.3	−	
ALL	T3D2	7.8	0.3	22.4	−
PLL	T3D2	10	0.3	7.0	−
LF	T3D2	15	0.3	14.1	−
CL	T3D2	7.5	0.3	10.5	−
ISL	T3D2	8	0.3	14.1	−
ITL	T3D2	10	0.3	0.6	−
SSL	T3D2	8	0.3	10.5	−
Bone graft	C3D4	450	0.3	−	−
Ti6Al4V	C3D10	110000	0.3	−	14452

*ALL, anterior longitudinal ligament; PLL, posterior longitudinal ligament; LF, ligamentum flavum; CL, capsular ligament; ISL, interspinous ligament; ITL, intertransverse ligament; SSL, supraspinous ligament.*

### Surgery Simulation and Biomechanical Evaluation

Four constructs with anterior column reconstruction and posterior fixation were created at the T7−T11 level: (B+P) anterior column reconstructed with a bone graft (rib or iliac bone) and posterior fixation with seven pedicle screws (Ti6Al4V) (this initial posterior fixation plan was made by two spinal surgeons according to the degree of vertebrae damage); (M+P) anterior column reconstructed with titanium alloy mesh (Ti6Al4V) and a bone graft and posterior fixation with seven pedicle screws; (M+P −T8) the right T8 pedicle screw was removed according to the second design; and (M+P+T9) the left T9 pedicle screw was added according to the second design (Figures 2B–E). For anterior column reconstruction, bone graft (rib or iliac bone) and mesh with bone graft were two commonly used method in treating spinal tuberculosis. The posterior fixation plans were mainly created to compare the stress difference of screws and rods, to avoid stress concentration or deficiency of implants, and so as to determine the necessary implants. To reconstruct the anterior column, the right facet joint at the T9–10 level and the right eighth rib were partially removed, and debridement was performed to create an appropriate site to fit the bone graft or titanium alloy mesh. The interfaces of the implants and bone were assigned with complete fusion. The 3D printed model was used to simulate the optimal surgical design according to the results of the biomechanical evaluation (Figure 2F).

### Loading and Boundary Conditions

In order to simulate the body weight (total body weight: 77 Kg) and thoracic spinal motions, a 400-N vertical axial pre-load and a 10-N⋅m moment were imposed on the superior portion of T7 in flexion and extension loading condition, respectively (Sim et al., 2010; Xiao et al., 2012; Arnold and Friis, 2015). The loading conditions were similar to the conditions used in previous lumbar FEA for the purpose of simulation and analysis (Wang B. et al., 2019). For all models, the boundary conditions imposed were set with the nodes on the inferior endplate of T11 constrained in all directions. The inferior and superior boundaries of the implants including the bone graft and titanium alloy mesh were constrained to the interface. Considering the physical movement limitations of the thoracic spine, the vertical axial load and motions of flexion and extension were selected for analysis. The total ROM was evaluated between different constructs. The von Mises stress on the implants was calculated and compared. Similar to previous researches, the max von Mises stress at bones near the implants was analyzed, in purpose of estimating the difference of stress distribution and the breakage and subsidence risk of implants (Lu and Lu, 2019; Tsouknidas et al., 2020).

### Model Verification and Validation

In order to get accurate results, the mesh grid of this patient-specific model was verified. The mesh convergence test was conducted with every model entity separately. Determination of the optimum mesh density is achieved with a mesh independent grid, ensuring that coarsening of the mesh does not disturb the stress field by more than 2%. The element type was first order element, and the elements’ quality was assessed according to average element quality (0.8505). The thoracic vertebrae finite element model with anterior column reconstruction and posterior fixation was of 574977 nodes and 357901 elements.

To validate the rationality of the model, T7−T8 and T10−T11 models were separated from the preoperative model. Same loading conditions were imposed on the superior portion of vertebrae in flexion, extension, lateral bending, and axial torsion. ROM of T7−T8 and T10−T11 were measured and compared with previous study separately (Panjabi et al., 1976; Ottardi et al., 2016; Zhang et al., 2020). In addition, the stress of rods and screws in postoperative models were compared with data from similar literatures (Alizadeh et al., 2013; Wang W. et al., 2019).

### Management of the Patient

The indications for surgery were ineffective conservative treatment, intractable back pain, progressive neurologic deficits, spinal instability, large paravertebral abscess and sequestrum formation, and the exacerbated destruction of thoracic vertebral body. Prior to the surgery, anti-tuberculosis drugs (rifampicin, 15 mg/kg; isoniazid, 5 mg/kg; ethambutol, 25 mg/kg; and pyrazinamide, 25 mg/kg) were administered for 2–3 weeks until the erythrocyte sedimentation rate (ESR) and C-reactive protein (CRP) were stable or started to decrease and other examination results and the patient’s condition were suitable for surgery. During medication, finite element models were simulated and analyzed, and 3D model was reconstructed and printed. The patient-specific preoperative surgical design, according to the biomechanical analysis, was prepared for implementation.

The patient was placed in a prone position after undergoing general anesthesia, with the upper limbs held upward. The damaged vertebrae were positioned under the intraoperative C-arm fluoroscope. A median incision of appropriate length was made, and the spinous process, bilateral lamina, facet joints, transverse process, and part of the ribs that needed to be excised were exposed. Transpedicular screws were inserted into the vertebrae according to the preoperative design. After screw placement, a temporary rod was placed on the side without rib excision to prevent movement during the debridement and the placement of the titanium alloy mesh. After excision of a part of the transverse process and rib, the collapsed vertebrae, necrotic disc, and prevertebral or paravertebral abscesses were completely removed. The prepared bone trough was cleaned by saline irrigation, and then, a suitable titanium mesh, with autogenic or allogenic bone, was inserted into the preoperatively designed site to reconstruct spinal stability. The position of the titanium mesh was confirmed by intraoperative fluoroscopy. An intact internal fixation instrument was placed according to the biomechanical evaluation. Isoniazid or streptomycin was placed locally, and two drainage tubes were placed prior to suturing.

The patient was treated with anti-tuberculosis drugs for 18 months. A thoracolumbar brace was continually used for 3 months postoperatively. The patient underwent physical examination; imaging examinations; and ESR, CRP, and hepatorenal function evaluation at later follow-ups. The kyphotic Cobb angles were measured in lateral thoracic radiograph. Loss of the Cobb angle was calculated at final follow-up. The loss of the Cobb angle and bone fusion were used to evaluate spinal stability. The postoperative effective spinal stability was indicated according to no complication of implants, no obvious change of the Cobb angle, and good bone fusion at final follow-up (Cui et al., 2013; Tang et al., 2019).

## Results

### Validation of the Models

**FIGURE 3:**
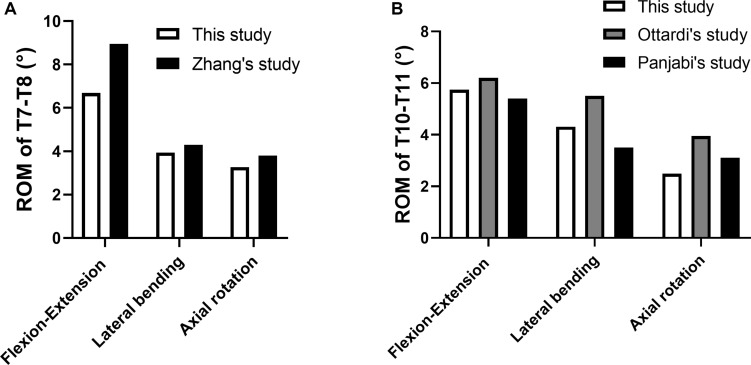
Comparison between the current models with previous studies. **(A)** ROM of T7−T8; **(B)** ROM of T10−T11.

In order to validate the models, the ROM of T7–T8 and T10–T11 under the same loading conditions were compared with previous studies under the same loading conditions, as shown in Figure 3 (Panjabi et al., 1976; Ottardi et al., 2016; Zhang et al., 2020). The stress values of screws obtained with surgical model (minimum: 25 MPa, maximum: 139 MPa) were of the same order of magnitude as those calculated in previous studies reporting approximate values [minimum: 26 MPa and maximum: 133 MPa from Alizadeh et al. (2013) and minimum: 70 MPa and maximum: 250 MPa from Wang W. et al. (2019)].

### von Mises Stress on the Implants

**FIGURE 4:**
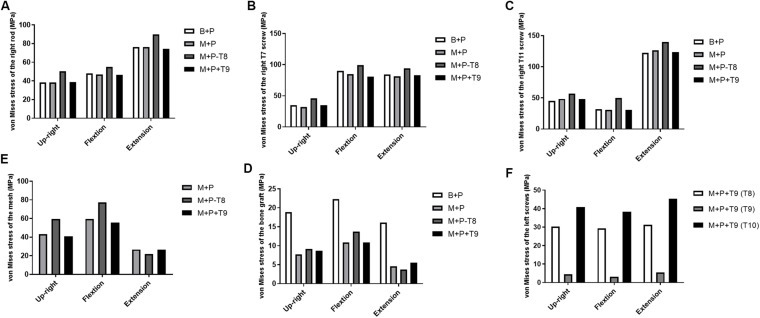
von Mises stress on the implants in different thoracic surgical constructs. **(A)** von Mises stress of the right rod; **(B)** von Mises stress of the right T7 screw; **(C)** von Mises stress of the right T11 screw; **(D)** von Mises stress of the mesh; **(E)** von Mises stress of the bone graft; **(F)** von Mises stress of the left screws.

The von Mises stress on the implants in the thoracic upright position and flexion and extension conditions are shown in Figure 4 and Supplementary Figure S1. Compared with the other three constructs, the von Mises stress on the right rod, T7 pedicle screw, and T11 pedicle screw increased in the upright position and flexion and extension conditions in M+P−T8 (Figures 4A–C). The highest von Mises stress on the titanium alloy mesh occurred under up-right, flexion, and extension conditions in M+P-T8 (Figure 4D). The von Mises stress on the bone graft was highest in B+P, but was decreased in M+P, M+P−T8 and M+P+T9 (Figure 4E). The von Mises stress on the left T9 pedicle screw in M+P+T9 was significantly lower than that on the left T8 and T10 pedicle screws, and the highest von Mises stress was only 5.42 MPa during extension (Figure 4F). In general, the constructs of M+P preserved the load-bearing right T8 pedicle screw and evaded the unnecessary left T9 pedicle screw.

### Max Stresses on the Bones

**FIGURE 5:**
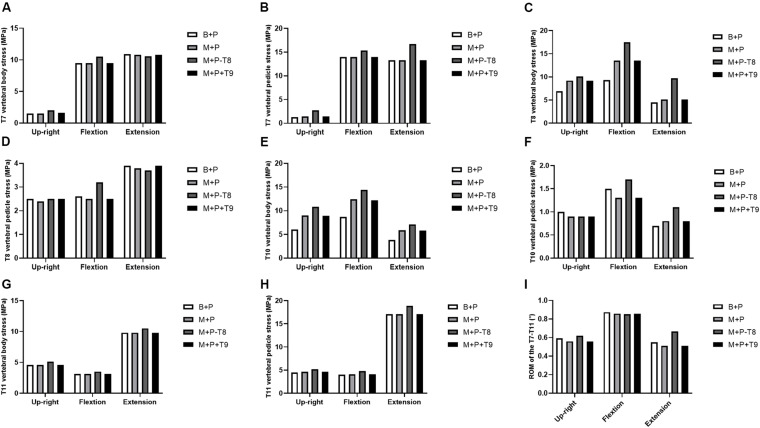
Max stress on bones near the implants and ROM of T7–T11. **(A)** T7 vertebral body stress; **(B)** T7 vertebral pedicle stress; **(C)** T8 vertebral body stress; **(D)** T8 vertebral pedicle stress; **(E)** T10 vertebral body stress; **(F)** T10 vertebral pedicle stress; **(G)** T11 vertebral body stress; **(H)** T11 vertebral pedicle stress; **(I)** ROM of the T7–T11.

The max stresses on the bones in the thoracic upright position and flexion and extension conditions are shown in Figure 5. The region with highest stress mainly distributed in the cortical bone near the implants. Compared with the other three constructs, the von Mises stress on the vertebral body near the right T7 pedicle screw increased in the upright and extension conditions in M+P−T8 (Figure 5A), and the von Mises stress on the vertebral pedicle near the right T7 pedicle screw increased in the upright position and flexion and extension conditions in M+P−T8 (Figure 5B). The von Mises stress on the bone (vertebral bodies of T8 and T10) near the mesh was highest in all loading conditions in M+P−T8, but decreased in B+P (Figures 5C,E). The von Mises stress on the vertebral pedicles of left T8 and T10 was highest in flexion condition in M+P−T8 (Figures 5D,F). Compared with the other three constructs, the von Mises stress on the vertebral body and pedicle near the right T11 pedicle screw increased in all loading conditions in M+P−T8 (Figures 5G,H). The von Mises stress on the bones near the implants had no obvious difference between M+P and M+P+T9.

### Range of Motion

Due to the bone defects of the anterior column, unstable vertebrae tilt was observed in the preoperative model under the upright position and flexion and extension conditions. All the surgical constructs restored the stability of the damaged thoracic spine. Compared with the preoperative model, ROM of the four surgical models were reduced under all loading conditions (Figure 5I). Compared with M+P, ROM increased slightly in B+P. Compared with the other three constructs, M+P−T8 had the largest ROM in the upright and extension conditions.

### Surgery Simulation and Assessment

Surgery was on the agenda after anti-tuberculosis drug treatment and biomechanical evaluation for 2–3 weeks. According to the biomechanical evaluation, anterior column reconstruction, with titanium alloy mesh and a bone graft, and posterior fixation, with seven pedicle screws, were found to be the optimal methods for the patient. Suitable pedicle screws were selected according to the patient-specific model. Surgery simulation with a suitable mesh was performed on the 3D printed model. Moreover, preoperative surgery simulation and explanation through the 3D printed model enhanced the understanding of the patient’s condition and facilitated patient-specific preoperative planning. Complete debridement, anterior column reconstruction, and posterior fixation were performed according to the preoperative M+P design.

### Postoperative Evaluation

**FIGURE 6:**
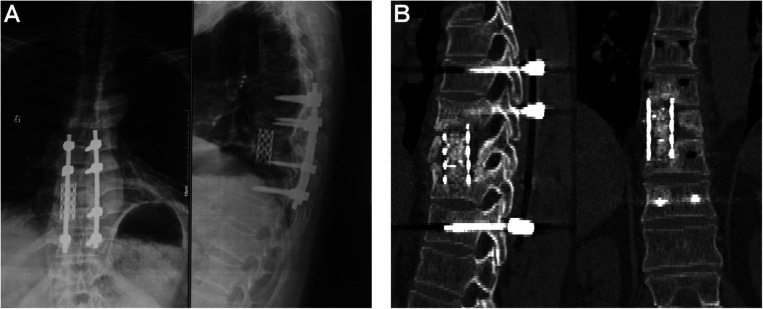
The radiographic and computed tomography images at the 2-year follow-up. **(A)** anteroposterior and lateral radiography; **(B)** CT images.

No complications, such as vascular injury, nerve injury, pleural damage, wound infection, and tuberculosis reoccurrence, were observed. After anti-tuberculosis drug treatment for 18 months, the patient was considered to be cured, according to normal physical and imaging examinations and ESR and CRP levels. The kyphotic Cobb angle preoperatively, before discharge, and at final follow-up was 32°, 29°, and 30°, respectively. The loss of Cobb angle was only 1°. According to the radiographic and CT images in Figure 6, bone fusion was achieved at the 2-year follow-up, and no implant breakage and displacement were observed.

## Discussion

The present study simulated and analyzed the biomechanical effects of the different methods of anterior column reconstruction and posterior fixation with the aim of optimizing patient-specific preoperative planning for thoracic spinal tuberculosis. Previous research studies demonstrated that the posterior fixation method, used in the correction of kyphosis and the maintenance of spinal stability, was superior to anterior fixation in the treatment of thoracic spinal tuberculosis (Huang et al., 2017; Shen et al., 2017; Wang et al., 2017; Wu et al., 2018). In the present study, instead of the fixation of two normal segments above and below the affected vertebrae, the fixation of posteriorly affected vertebrae was designed and simulated. According to previous studies, the clinical effectiveness and feasibility of the fixation of posteriorly affected vertebrae in treating thoracic spinal tuberculosis had been confirmed (Liang et al., 2018, 2019). However, complications including bone graft tilt, internal fixation loosening, and loss of the Cobb angle remain as unresolved issues after the fixation of posteriorly affected vertebrae (Liang et al., 2018). A possible reason might be the unbalanced stress condition of the internal implants in motion. A patient-specific surgical strategy would ensure that an optimal reconstruction and fixation approach, which will prevent stress concentration and implant imbalance.

The finite element models were reconstructed with the purpose of evaluating the stress on the implants preoperatively. It has been reported that the increased stress of implant was associated with the postoperative breakage and subsidence risk of implants (Zhang M. et al., 2016; Fan et al., 2019). The present study compared the von Mises stress on the rod, pedicle screws, bone graft, and mesh of the different surgical constructs. In purpose of analyzing the influence of internal implants only, perfect interfaces with bonded constraint were simulated between implants and bone. Compared with the other three constructs, the von Mises stress on the bone graft was highest in B+P. Previous biomechanical study had demonstrated that the increased stress distribution of intervertebral implants could increase the risk of implant subsidence and dislodgement based on long-term mechanical evaluation (Zhang M. et al., 2016). In present FEA, the increased stress distribution of bone graft may cause postoperative subsidence and displacement of bone graft. The von Mises stress on the vertebral bodies of T8 and T10 near the mesh increased in models with mesh. Moreover, the use of mesh could disperse stress and decrease the risk of postoperative complication. The biomechanical results proved that anterior column reconstruction with mesh and bone graft was superior to bone graft only. The posterior pedicle screw fixation could reduce anterior bone graft stress and the relative motion, which have beneficial influences on the bone fusion (Kim, 2007). Therefore, the effective spinal fixation is closely related to postoperative bone fusion.

Even though posterior fixation and fusion is an effective method for the treatment of spinal tuberculosis, postoperative breakage of rod or screws and loosening of screws remain clinical problem for the surgeons (Alam et al., 2015; Zhang P. et al., 2016; Liu et al., 2018). Since the lower part of the T8 vertebra as an affected vertebrae was found to be damaged, the M+P−T8 surgical model was simulated and analyzed in order to assess the loading condition of the right T8 pedicle screw. The von Mises stress on the right rod, right T7 screw, right T11 screw, and mesh increased after the right T8 pedicle screw was removed. In addition, the von Mises stress on the bones (vertebral bodies and pedicles) near the implants basically all increased as well. Previous finite element studies reported that the maximum von Mises stress of the bone surrounding internal implants was related to the risk of implants subsidence or screws loosening (Zhang et al., 2018; Lu and Lu, 2019). Moreover, compared with M+P and M+P+T9, ROM of T7–T11 increased slightly in M+P+T8. This revealed that the right T8 pedicle screw was essential for maintaining spinal stability and the M+P −T8 surgical construct might increase the breakage or loosening risk of screw and rod and the risk of mesh subsidence. Similar with previous studies, fixation of affected vertebrae could be effective to enhance the spinal stability (Liang et al., 2019). Moreover, the effectiveness of the right T8 pedicle screw prevented the upper normal vertebra from requiring fixation. The biomechanical analysis also showed that the von Mises stress on the bone graft increased in the B+P surgical model. This result suggested that anterior column reconstruction with the titanium alloy mesh and bone graft protects the bone graft from stress concentration and possibly prevents postoperative bone graft tilt or subsidence. With regard to the impact of the left T9 pedicle screw, since the von Mises stress was very small and there was no obvious difference of stress on the implants and the bones near the implants between M+P and M+P+T9, the insertion of the left T9 pedicle screw during surgery was unnecessary. FEA provided the biomechanical evidence to support the M+P surgical constructs as the optimal one. In brief, the M+P surgical model not only decreased the stress concentration of anterior bone graft and avoided stress concentration or deficiency of posterior implants, but also provided effective posterior fixation to maintain spinal stability. There was no postoperative complication mainly caused by unbalanced biomechanical effect, such as implant breakage or displacement, obvious loss of Cobb angle, and bone non-union. The postoperative clinical results indicated the effectiveness of surgical construct. However, the postoperative biomechanical effect was just assessed by radiographic data. Application of preoperative biomechanical evaluation in clinic should be clarified in more details with more clinical cases and longer follow-up time.

In addition, an assisted 3D printed model was reconstructed to elucidate the damaged thoracic vertebrae and used to simulate surgery preoperatively with the purpose of improving the patient’s comprehension of spinal tuberculosis and the posterior surgical approach. It had been reported that preoperative 3D models can help surgeons plan operations, assist young doctors in learning surgical methods, and enhance communication between patients and surgeons (Liu et al., 2020). Moreover, preoperative 3D printed models, surgical guide plates, and customized implants in orthopedic clinical application exhibited significant advantage, including the individual customization, rapid manufacturing, and efficient intraoperative drilling and osteotomy (Zhang et al., 2019a, b; Dong et al., 2020). In present study, assisted 3D printed model showed the focus of thoracic tuberculosis and the destruction of vertebral bodies directly, which could enhance the comprehension of spinal tuberculosis and the necessity of surgical treatment. The surgical simulation and explanation of selected M+P surgical construct improved learning effect of young doctors and patients’ approval of surgical plan. In present study, the biomechanical evaluation of the FEA and the assisted 3D printed model for patient-specific preoperative planning was the first combined application used for the management of thoracic spinal tuberculosis. The postoperative effective spinal stability was indicated according to no complication of implants, loss of the Cobb angle (1° in present study), and good bone fusion at final follow-up. Overall, our results suggested that M+P was sufficient for maintaining spinal stability in treating thoracic spinal tuberculosis in this patient, and the clinical results indicated the effectiveness of preoperative surgical plan. This combined application of finite element and 3D printed models may provide a potential and effective method for patient-specific preoperative planning.

There are some limitations to the present study. First, similar to many individual models, it is difficult to fully validate the finite element models due to the limited clinical or experimental data and standardized methods (Jones and Wilcox, 2008; Galbusera et al., 2015; Robinson et al., 2018). In the present study, simulations only focused on the difference in implant stress under flexion-extension conditions and emphasized the physical soundness of conceptual models rather than direct or indirect validation. Second, the models were simplified and idealized due to the complexity of the spinal structure (active muscles and facet joints) and individual variations, which could only reflect the biomechanics objectively. Live contracting muscles and ribs as potential factors, which may influence the biomechanical evaluation, were simplified and omitted. Moreover, the interface conditions of the implants and bone were simulated as complete fusion, so this model cannot be used to study the immediate postoperative period. Third, changes in the position and orientation of the pedicle screws and titanium alloy mesh may alter the stress distribution, which was hardly considered during the operation. Fourth, the influence of tuberculosis on the elasticity modulus of the adjacent vertebrae and ligaments was quite challenging to be taken into account. Finally, effectiveness of preoperative surgical plan was assessed by postoperative radiographic results without direct or indirect biomechanical assessment. The combination of the clinical data from a large number of patients with long-term follow-up and the FEA may be more reliable.

However, the findings of the FEA reduced our dependence on animal and cadaveric experiments and were considered a significant complement to clinical studies. Moreover, this study showed that FEA and 3D printed model were tools that could be extremely useful and effective in the preoperative planning. The biomechanical evaluation using FEA provided preoperative stress distribution, which could be used to assess the effective and stability of selected surgical approach. Assisted 3D printed model improved understanding of thoracic tuberculosis and preoperative surgical plan. Future clinical studies should be conducted, and true animal or human models should be analyzed and evaluated to verify the results of the FEA.

## Conclusion

In conclusion, the biomechanical behavior of four surgical constructs in the thoracic spinal tuberculosis of T7–T11 was compared using FEA. We recommend M+P as the optimal preoperative planning method as it enabled the preservation of the normal vertebra and prevented unnecessary internal fixation. The assisted 3D printed model is likely to provide potential technology in the preoperative planning for thoracic spinal tuberculosis, which can facilitate preoperative surgical simulation and improve the understanding of the patient’s condition.

## Data Availability Statement

The raw data supporting the conclusions of this article will be made available by the authors, without undue reservation.

## Ethics Statement

The studies involving human participants were reviewed and approved by The Ethics Committee of Tongji Medical College, Huazhong University of Science & Technology. The patients/participants provided their written informed consent to participate in this study.

## Author Contributions

BW and WK designed the study, analyzed the data, and wrote the manuscript. WH participated in the design of the study and analyzed the data. XZ and CY collected the clinical data, helped in writing the manuscript, and instructed on the surgical technique. All authors read and approved the final manuscript.

## Conflict of Interest

The authors declare that the research was conducted in the absence of any commercial or financial relationships that could be construed as a potential conflict of interest.
