# Supplementary figures and images for: Biomechanical Evaluation and the Assisted 3D Printed Model in the Patient-Specific Preoperative Planning for Thoracic Spinal Tuberculosis: A Finite Element Analysis
Source: Front Bioeng Biotechnol. 2020 Jul 17;8:807. doi: 10.3389/fbioe.2020.00807 (PMC7379841; doi:10.3389/fbioe.2020.00807)

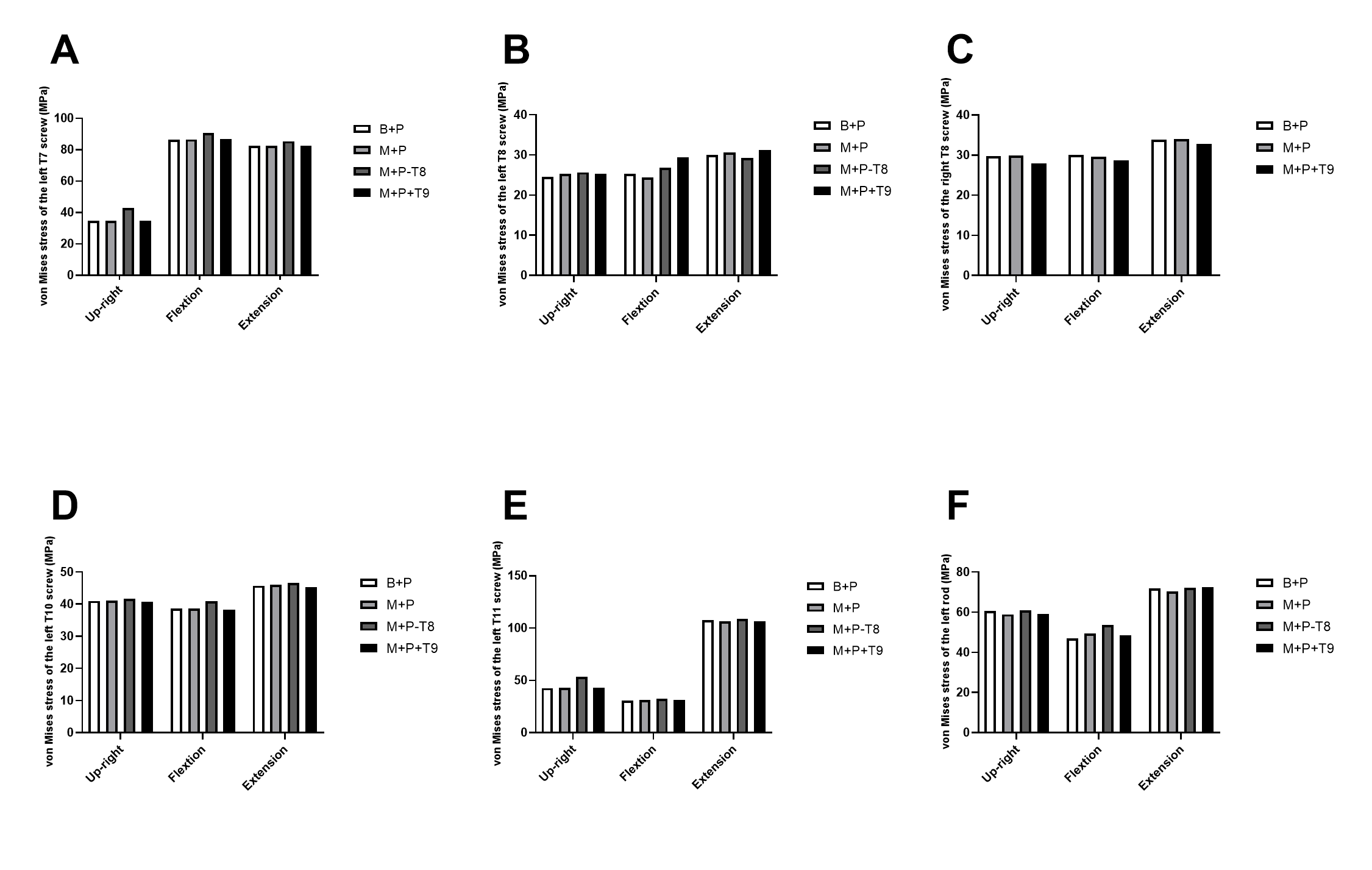

Supplement: Supplementary file 1 [file Image_1.TIF]
